# Harnessing genomics to improve health in the Eastern Mediterranean Region – an executive course in genomics policy

**DOI:** 10.1186/1478-4505-3-1

**Published:** 2005-01-21

**Authors:** Tara Acharya, Mohammed Abdur Rab, Peter A Singer, Abdallah S Daar

**Affiliations:** 1 Joint Center for Bioethics, University of Toronto, 88 College Street, Toronto, ON – M5G 1L4, Canada; 2 World Health Organization, Eastern Mediterranean Regional Office, Cairo, Egypt; 3 Faculty of Medicine, University of Toronto, 1 King's College Circle, Toronto, Ontario – M5S 1A8, Canada; 4 Department of Public Health Sciences, University of Toronto, 12 Queen's Park Crescent W. Toronto, ON – M5S 1A8 Canada; 5 Department of Surgery, University of Toronto, Banting Institute, 100 College Street, Toronto, ON – M5G 1L5 Canada; 6 The McLaughlin Centre for Molecular Medicine, University of Toronto, 620 University Avenue, Toronto, ON – M5G 2C1 Canada

## Abstract

**Background:**

While innovations in medicine, science and technology have resulted in improved health and quality of life for many people, the benefits of modern medicine continue to elude millions of people in many parts of the world. To assess the potential of genomics to address health needs in EMR, the World Health Organization's Eastern Mediterranean Regional Office and the University of Toronto Joint Centre for Bioethics jointly organized a Genomics and Public Health Policy Executive Course, held September 20^th^–23^rd^, 2003, in Muscat, Oman. The 4-day course was sponsored by WHO-EMRO with additional support from the Canadian Program in Genomics and Global Health. The overall objective of the course was to collectively explore how to best harness genomics to improve health in the region. This article presents the course findings and recommendations for genomics policy in EMR.

**Methods:**

The course brought together senior representatives from academia, biotechnology companies, regulatory bodies, media, voluntary, and legal organizations to engage in discussion. Topics covered included scientific advances in genomics, followed by innovations in business models, public sector perspectives, ethics, legal issues and national innovation systems.

**Results:**

A set of recommendations, summarized below, was formulated for the Regional Office, the Member States and for individuals.

• Advocacy for genomics and biotechnology for political leadership;

• Networking between member states to share information, expertise, training, and regional cooperation in biotechnology; coordination of national surveys for assessment of health biotechnology innovation systems, science capacity, government policies, legislation and regulations, intellectual property policies, private sector activity;

• Creation in each member country of an effective National Body on genomics, biotechnology and health to:

- formulate national biotechnology strategies

- raise biotechnology awareness

- encourage teaching and training of biotechnology

- devise integration of biotechnology within national health systems.

**Conclusion:**

The recommendations provide the basis for a road map for EMR to take steps to harness biotechnology for better and more equitable health. As a result of these recommendations, health ministers from the region, at the 50th Regional Committee Meeting held in October 2003, have urged Member States to establish national bodies of biotechnology to formulate a strategic vision for developing biotechnology in the service of the region's health. These efforts promise to raise the profile of genomics in EMR and increase regional cooperation in this exciting new field.

## Background

In a recent study, University of Toronto researchers identified the "Top 10 Biotechnologies to Improve Health in Developing Countries" [1]. The study underscores the importance of harnessing new technologies to improve global health and development, a belief that is gaining widespread acceptance. For instance, the overall goal of the United Nations Millennium Project's Science and Technology Task Force is to address how science and technology can be leveraged to help countries achieve the Millennium Development Goals (MDGs) [2]. Its mission is guided by the understanding that most of the MDGs cannot be reached without a strong contribution from science and technology. The potential contribution of genomics and biotechnology to these goals has also been demonstrated [3]. However, these technologies are most beneficial to countries that have the scientific capacity to absorb and use them. The aim of the Inter-Academy Council on Science and Technology Capacity (IAC) was to develop a global strategy for promoting capacities in science and technology and the first report of the Council was recently presented to UN Secretary General Kofi Annan [4].

There is a wide range of scientific capacity and health system development across the Eastern Mediterranean Region (EMR) [5], which consists of 21 countries. Some countries in the region have taken the initiative in biotechnology by establishing regulations and encouraging private sector involvement. In other countries of the region, there is a serious lack of scientific capacity not only to conduct research and development in biotechnology but even to absorb the benefits of biotechnology and apply them to help meet the health and socio-economic needs of the population [6]. According to UNDP's Arab Human Development Report 2003, research and development in the Arab world represents less than 0.2% of Gross National Product (GNP). Fewer than one in 20 Arab university students were pursuing scientific disciplines, compared, for instance, to one in five in South Korea [7]. There is a risk that, as the genomics revolution gathers momentum, the imminent genomics divide between developed and developing countries will increase unless urgent action is taken to reverse the trend [8].

In order to assess the potential of genomics to address health needs in the Region, the World Health Organization-Eastern Mediterranean Regional Office (WHO-EMRO) and the University of Toronto Joint Centre for Bioethics jointly organized a Genomics and Public Health Policy Executive Course, held September 20^th^–23^rd^, 2003, in Muscat, Oman. This 4-day course/workshop was sponsored by WHO-EMRO with additional support from the Canadian Program in Genomics and Global Health.

The overall objective of the Genomics Policy Executive Course was to familiarize participants with the potential of genomics and related biotechnologies to address health needs and to collectively address the question of how best to harness genomics to improve health in the region (table 1).

**Table 1:** Objectives of the Genomics Policy Executive Course

To familiarize participants with the current status and implications of health genomics/biotechnology, and to provide information relevant to public policy on health genomics/biotechnology
To provide frameworks for analyzing and debating the policy issues and related ethical questions in health genomics/biotechnology, and to help understand, anticipate and possibly influence the legal and regulatory frameworks under which health biotechnology industries will operate, both nationally and internationally
To begin developing an opinion-leaders network across different sectors (industry, academic, government, NGOs) by sharing perspectives and building relationship
To formulate recommendations for future policy and strategic directions at the regional, national and individual levels.

## Methods

We based the program and designed the sessions and lecture topics of this course on prior courses held in Nairobi, Kenya in March 2002; in Toronto, Canada in May 2002; and in Kumarakom, India in January 2003. The sessions and presenters of the course are shown in table 2. The course participants and facilitators for each session were identified through a combination of recommendations from field experts in the region and from WHO's network of experts. People identified to participate in the course included scientists from academic institutions and industry, industry executives, regulatory officials, representatives from the legal sector, and media. The participants were carefully chosen in an attempt to represent a wide range of interests relevant to the emerging area of genomics and to have geographical, discipline and gender balance. They included scientists from academic institutions and industry, industry executives, legal and regulatory officials, WHO representatives and the media. In total there were 51 participants, from 13 countries of EMR. We drew as many of the faculty as possible from the region.

**Table 2:** Program

**Saturday, 20 September 2003**	
**08:00 – 08:30**	Registration
**08:30 – 10:30**	Session I: Opening Address *H.E. Dr Ali Bin Moosa, Minister of Health, Oman* *Dr H. A. Gezairy, Regional Director, EMRO* Introduction and Course Overview *Team from University of Toronto*
	Session Chair: *Dr Ali Jaffer Suleiman, Ministry of Health, Oman*
**10:30 – 11:00**	Coffee break
**11:00 – 12:30**	Genomics: Scientific Developments Professor Riad Bayoumi, Sultan Qaboos University
**12:30 – 14:00**	Lunch Break
**14:00 – 15:30**	Session III: WHO Report on Genomics and World Health *Professor Alexander Capron, Director, Department of Ethics, Trade and Human Rights, WHO/HQ*
**15:30 – 16:00**	Coffee break
**15:45 – 16:30**	Session IV: Top 10 Biotechnologies for Improving Health in Developing Countries *Professor Abdallah S. Daar, University of Toronto*

**Sunday, 21 September 2003**	

**08:30 – 09:00**	Golden Nuggets (previous day's summary) *Dr Peter A. Singer, University of Toronto*
**09:00 – 10:30**	Islamic Perspective on Stem Cells, Cloning, Genetic Engineering, ..etc *Dr Mohammed Al Bar, King Fahd Medical Research Centre, Saudi Arabia*
**10:30 – 11:00**	Coffee break
**01:00 – 12:30**	Intellectual Property Rights Professor Richard Gold, McGill University, Centre for Intellectual Property Policy
**12:30: – 14:00**	Lunch Break
**14:00 – 15:30**	Business Models *Mr Khalil Ahmed, Managing Director, Shantha Biotech, India* *Dr Peter A. Singer, University of Toronto*
**15:30 – 16:00**	Coffee break
**16:00 – 17:30**	Group Work

**Monday, 22 September 2003**	

**08:30 – 09:00**	Golden Nuggets (previous day's summary) *Dr Peter A. Singer, University of Toronto*
**09:00 – 10:30**	Innovation Systems *Dr Peter A. Singer, University of Toronto*
**10:30 – 11:00**	Coffee break
**11:00 – 12:30**	Regulatory Systems and Related Issues *Dr D.C. Jayasuriya, Director, UNAIDS, Pakistan* *Dr Anwar Nasim, Chairman, National Council of Biotechnology, Pakistan*
**12:30 – 13:00**	TRIPS and Pharmaceutical Issues in Public Health. *Dr. Abdel Aziz Saleh Special Advisor to the Regional Director for Medicine, WHO/EMRO*
**13:00 – 14:00**	Lunch Break
**14:30 – 15:30**	Public Engagement *Mr Ehsan Masood, Scidev.net, United Kingdom*
**15:30 – 16:00**	Coffee Break
**16:00 – 17:30**	Group Work

**Tuesday, 23 September 2003**	

**08:30 – 09:00**	Golden Nuggets (previous day's summary) *Dr Peter A. Singer, University of Toronto*
**09:00 – 10:00**	Opinion Leaders Network *Dr Peter A. Singer, University of Toronto, Dr Tara Acharya, University of Toronto*
**10:00 – 10:30**	Break
**10:30 – 12:30**	Group Work
**12:30 – 14:00**	Lunch break
**14:00 – 15:30**	Group Presentations and Discussion
**15:30 – 16:00**	Coffee Break
**16:00 – 17:30**	Recommendations, Concluding Remarks and Closure of the Workshop *Dr Peter Singer, University of Toronto*

The sessions dealt with a wide range of relevant topics, starting with recent scientific advances in genomics and stem cell research, followed by discussions on business models in genomics and biotechnology, intellectual property rights and regulatory frameworks, public engagement and an internet-based opinion leaders' network. The presentations were designed to be interactive and foster active discussion from, and among, the participants, and each presentation was followed by a moderated discussion period. Early in the course, the attendees were placed into one of five study groups – these groups were carefully designed to capitalize on the diverse backgrounds of the participants. Each group was assigned the task to on the deliberate the key question **"How best to harness genomics and biotechnology to improve the health of the people in the Eastern Mediterranean Region?" **The groups met frequently to discuss the presentations, and each participant was also provided a course reader with additional literature on the lecture topics (table 3). The overall task of these study groups was to draw upon the course material and their own experiences and propose a set of recommendations for genomics and biotechnology policy in the region. On the last day of the meeting each group presented these recommendations.

**Table 3:** Course Readings

Bhutta ZA (2002) Ethics in international health research: a perspective from the developing world. *Bull World Health Organ*.;80(2):114–20. Review.
Bloom BR & Trach D (2001) Genetics and Developing Countries. *BMJ*;322:1006–7.
Capron AM (2001) Stem Cells: Ethics, Law and Politics. Biotech Law Report;5:678–699.
Collins FS, Green ED, Guttmacher AE, Guyer MS; US National Human Genome Research Institute. (2003) A vision for the future of genomics research. Nature.24;422(6934):835–47.
Daar AS, Thorsteinsdóttir H, Martin DK, Smith AC, Nast S, Singer PA. (2002) Top ten biotechnologies for improving health in developing countries. *Nat Genet*.;32(2):229–32.
Gold ER (2003) SARS genome patent: symptom or disease? *Lancet*.;361(9374):2002–3.
Juma C & Konde V (2002). The New Bioeconomy: Industrial and Environmental Biotechnology in Developing Countries. Geneva, Switzerland: United Nations, July 2002.
Lundvall B, Johnson B, Andersen EA, Dalum B (2002) National systems of production, innovation and competence building. *Research Policy*;31:213–231
Nasim A (2000) Ethical Issues of the Human Genome Project: An Islamic Perspective *in *Bioethics in Asia. Eubios Ethics Institute Eds: N Fujiki and DRJ Macer p. 209–214
Pang T & Weatherall D (2002) Genomics and Global Health. *BMJ*;324:p.1051–52
Singer PA, Daar AS (2001) Harnessing genomics and biotechnology to improve global health equity. *Science*;294(5540):87–9.
Sulston J (2003) Beyond release: the equitable use of genomic information. *Lancet*.;362(9381):400–2.
Thorsteinsdóttir H, Daar AS, Smith RD, Singer PA (2003) Genomics – a global public good? *Lancet*.;361(9361):891–2.
Time to Unite Islam and Science. *Nature*. 2003 Mar 13;422(6928):99.

In order to assess the level of interest in the formation of an email-based opinion leaders' network to continue discussion among the participants following the course, a brief survey was conducted on the participants' internet access and their expectations of the network.

## Results

Dr Peter A. Singer, Director of the University of Toronto Joint Centre for Bioethics, described the overall aim of the course "How to best harness genomics to improve health in the region", as well as the 4-day program. Dr Ali Jaffer Suleiman of the Ministry of Health in Oman acted as chairperson of the meeting.

Professor Riad Bayoumi of Sultan Qaboos University highlighted new scientific developments that have resulted from the genomics revolution, such as proteomics; mapping of single nucleotide polymorphisms (SNPs) to understand human genetic variation and its relationship with disease; gene expression chips to monitor differential gene expression and identify drug targets; and bioinformatics as a new field that combines biology, mathematics, statistics and computer programming to mine large-scale biological data.

Professor Alexander Capron, Director, Department of Ethics, Trade and Human Rights at WHO, described the 2002 report of the World Health Organization "Genomics and World Health" [9]. He called attention to the recommendations from the report – these include improving technical cooperation between WHO and its member states (e.g. assessing the health impacts of genomics research to support informed priority setting; capacity building for genomics research and biotechnology in developing countries; development of ethical review structures and bioethics capacity).

Professor Abdallah Daar described a recent study, conducted by the University of Toronto Joint Centre for Bioethics' Canadian Program on Genomics and Global Health, to identify the ten most promising biotechnologies for improving health in developing countries in the next five to ten years. These technologies offer guidance to those who can influence the direction of R&D in developing countries and challenge common assumptions about the relevance of biotechnology for these countries, as shown by the mapping of the Top 10 Biotechnologies onto the UN Millennium Development Goals [10]. However, to foster biotechnology in developing countries it is essential to build capacity (among researchers, politicians, legislators, entrepreneurs, etc).

Professor Al Bar of King Fahad University, Saudi Arabia, illustrated Islamic perspectives on genetic testing, cloning, recombinant DNA technology and other genomics-related technologies. He showed that while Islam and science have always been aligned through history, today's religious leaders in the region must take the initiative to develop and formulate recommendations for genomics.

Dr. Peter Singer described the concept of innovation systems and presented some observations of other countries' innovation systems and their successes. One definition of a national system of innovation (NSI) is the "network of institutions in the public and private sector whose activities initiate, import, modify and diffuse new technologies" [11-13]. The application of NSI to developing countries is a fairly recent concept. The Canadian Program on Genomics and Global Health is currently conducting studies of the NSI of 7 developing countries: Brazil, China, Cuba, Egypt, India, South Africa and South Korea [14]. Despite the identification of factors that foster innovation systems, there is no one model that guarantees success – each country follows its own unique path.

Mr. Khalil Ahmed, Managing Director of Shantha Biotechnics, described the successes of this biotechnology company based in Hyderabad, India. Shantha was established in 1993, in part with seed money from the Government of Oman. Today, it is the first Indian company to receive WHO certification for a recombinant hepatitis-B vaccine Shanvac-B™, paving the way for UNICEF to buy 8.5 million doses for distribution globally. Hepatitis B vaccine is currently priced internationally as high as $8–10 per dose, while Shantha is selling it at $2 per dose [15,16].

Professor Richard Gold of McGill University's Centre for Intellectual Property Policy described the basics of intellectual property rights, patents and copyright issues, as well as policy options for developing countries in the international context. He outlined the characteristics of international agreements, which offer considerable flexibility to countries on how to apply patent laws to genomics and biotechnology. Developing countries have a number of options, such as compulsory licensing and research exemptions, to deal with property rights.

Dr Jayasuriya, Director of UNAIDS in Pakistan described ways in which legal systems can facilitate best use of biotechnology. It is essential that legal reform keep up with the rapidly evolving science of genomics. Health law can facilitate best use of biotechnology – by providing for fast-track approval of biotechnology products; reducing import duties on health interventions; and allowing multiple channels of procurement and distribution to improve access and optimize prices.

Dr Anwar Nasim, Chairman of Pakistan's National Council of Biotechnology, described the role of regulations both to promote useful biotechnologies and limit their risks for human health and the environment. A national-level regulatory body could provide guidelines for the use and release of biotechnology products, conduct biosafety reviews and risk assessments and formulate feedback mechanisms to improve the system through experience. The National Commission of Biotechnology of Pakistan, established in November 2001, focuses on biotechnology regulatory issues in health, agriculture, environment and industry. Similar commissions could be set up in other countries of the region and their interaction could further regional cooperation in biotechnology.

Mr. Ehsan Masood of Scidev.net pointed out that active public engagement based upon knowledge can stimulate action to improve public health. Public engagement is far greater in today's world than it has ever been in the past. This is because of several factors including; a) the growing implications of research on public health, b) the increased awareness in civil societies to invest in health care and research, and to influence policy change and action, c) development and access of information technologies.

On the last day of the course the five participant groups, who had been assigned the task to on the deliberate the key question **"How best to harness genomics and biotechnology to improve the health of the people in the Eastern Mediterranean Region?"**, were invited to present their findings. The main points of these group presentations are summarized in the recommendations that emerged from the course.

## Discussion

The session discussions and group presentations underscored the urgent need for action to create the enabling environments (at regional, national and individual levels) for research and development in genomics and biotechnology.

The issue of awareness of biotechnology among political leaders to garner support at the highest level was raised early on in the course, and was reinforced throughout the course discussions. The participants felt strongly that political commitment is crucial to the advancement of biotechnology in the region. Political leadership is a critical factor in raising the profile of science in developing countries. A prominent example in the Eastern-Mediterranean Region is that of the Sultan Bin Mohammed Al-Qassimi, Shaikh of the Arab emirate of Sharjah. He has made a dedicated effort to change the face of science in the Gulf [17]. For example, in an attempt to attract Arab scientists working abroad to return to work in the Gulf, and more specifically to Sharjah, he has built two universities, six museums and established a science foundation in just a decade. He is also actively engaged in creating an environment of regional cooperation.

The course attendees recommended that WHO-EMRO should take the responsibility to engage political leaders in the region's national governments. The participants shared experiences of the importance of government support for biotechnology – in Egypt there appears to be relatively strong support for biotechnology by the government, with biotechnology activity in academic and other research institutions as well as in the private sector. In Iran, considerable advances have been made in private and academic research centres, leading to a number of biotechnology products that are soon to reach the market, as well as well-respected scientific journals such as the Iranian Journal of Biotechnology [18]. Other countries such as Pakistan, Tunisia, Lebanon, and Morocco are making steady gains in biotechnology, some in conjunction with their powerful public health sectors and others as a result of their strong scientific bases.

It will also be crucial to involve, inform and engage religious leaders in the region in order to promote genomics and biotechnology for improving public health. In doing so, it is worthwhile to note that Islam and science have always been aligned through history and it is well-established that Islam has historically contributed tremendous achievements to the advancement of science. [19] A recent article argues that the Muslim world has neglected to pay attention to contemporary ethical issues in science and technology [20]. The author calls for the establishment of an independent Islamic bioethics panel to advise Islamic governments and communities. Other measures, such as training Muslim bioethicists, incorporating biomedical issues into school curricula and educating the community about such issues are also recommended.

The active participation and involvement of organizations like COMSTECH was recommended to give support and guidance to WHO-EMRO's efforts. COMSTECH was established by the Islamic Summit in 1981 and includes among its objectives the building of indigenous capabilities in the fields of science and technology, promotion and continuing cooperation and coordination in scientific and technological areas of is member states and creation of effective institutional structure for planning research, development and monitoring of scientific and technological activities. COMSTECH has already launched networks across the region for exchange of information, and has valuable experience in the promotion of cooperation and coordination amongst the member states in science and technology activities in high technology areas [21].

A major discussion point at the workshop was the effective assessment of existing capacity in the region. Several participants shared disappointment over the relative lack of participation of the region in global science and technology advances, citing, for instance, poor representation of the region at international scientific conferences. Many felt the need to evaluate and assess the level of scientific activity and capacity in individual countries in the region, in order to identify, among other things, strengths and weaknesses, entry points for countries, opportunities for regional collaboration, and areas for improvement. The workshop attendees felt it important to carry out this type of survey of each country's innovation system as a prerequisite to revising and revamping national and regional genomics policy. The factors identified by the participants as important for assessment in this survey – scientific capacity within public and private sector, private enterprise, religious and political leadership and intellectual property rights – can be considered to be part of the National System of Innovation (NSI). This proposed survey of NSI in the region can help to identify factors for successful growth of biotechnology sectors. Similarly, the recently completed study by the Canadian Program on Genomics and Global Health of the health biotechnology innovation systems of Brazil, China, Cuba, Egypt, India, South Africa and South Korea may also help to identify some of these factors [22]. Workshop participants from Egypt, Iran, Lebanon, Pakistan, and Tunisia contributed to the discussion of NSI with descriptions of components of their countries' biotechnology innovation systems. In Egypt, there is increased recognition of the importance of intellectual property rights in fostering innovation. The Minister of Health has taken a keen interest in improving linkages between sectors to enhance inter-sectoral communication. The president himself was instrumental in the establishment of the Mubarak City for Scientific Research and Technology (MCSRT), which is currently engaging in collaborative projects with the United States and with China. Private sector development is of high priority, as indicated by government support of companies such as Vacsera, which is now making recombinant insulin in Egypt [23]. One successful story from Egypt is the development by scientists at the Agricultural Genetic Engineering Research Institute in Giza of a powerful bio-pesticide based on a new strain of *Bacillus thuringiensis *[24]. In Iran, the trade embargo has led to a shortage of funds, but has in some ways helped to strengthen the innovation system through the need for self-reliance. Iran and Cuba have reached an agreement for cooperation and transfer of technology to produce hepatitis-B vaccine, interferon-α, streptokinase, and erythropoietin. Iran has developed several products based on recombinant DNA technology, and the country is also actively building research collaboration networks. Pakistan has had some successes in agricultural biotechnology and made some advances in bioethics. Although there are powerful institutes in place, the country needs to continue to strengthen facilities, resources and human capital. There is a need to bring products to the public. Lebanon has a well-developed healthcare industry, and has developed expertise in genetic testing, bioethics as well as intellectual property rights. Tunisia has strong research infrastructure and has made advances in genetic counseling, cytogenetics, and diagnosis of genetic diseases, along with regulation and legislation. However the pharmaceutical industry is not able to optimize the potential of public sector research, for which regional cooperation would be valuable. The participants identified strengthening the linkages between academia and the private sector as key to strengthening capacity in genomics. One example of a concerted effort to improve academic-private sector links is Jeddah BioCity, a research facility with close ties to the King Faisal Specialist Hospital and Research Center. Founded by Sultan Bahabri, head of King Faisal Specialist Hospital and Research Center in Jeddah and other Saudi scientists, this private venture plans the construction of state of the art biotechnology laboratories and companies and is intended to make Saudi Arabia a world leader in biotechnology.

As highlighted by the 2004 report of the UN Commission on Private Sector and Development report [25], the process of commercialization for development involves the dissemination and facilitation of knowledge flows between public and private sectors of both developed and developing markets. The report recommends action in both the public and private spheres, and emphasizes the linkages between these spheres, recognizing the importance of cooperation and partnerships to achieve goals. With stronger public-private linkages, the private sector in developing countries will be able to help provide new genomics-based technologies at affordable prices. Given that over the last few decades, market forces have driven the R&D agenda of pharmaceutical companies based in the North to de-emphasize the health concerns of developing countries, it is becoming increasingly important to develop indigenous capacity in private enterprise. The successes of biotechnology firms in developing countries such as India and Egypt were seen as a source of inspiration by the course attendees.

The attendees observed that the key to effective participation in the genomics revolution is building scientific capacity, and expressed serious concern over the region's limited capacity to absorb and utilize genomics and biotechnology. Critical to capacity-building is access not just to technology but, more importantly, to scientific knowledge. One point-of-entry that was greeted with enthusiasm by the participants was bioinformatics, which is typically less-resource intensive compared with other genomics-related sciences, such as sequencing and proteomics. According to the report on the "Top 10 technologies to improve health in developing countries" countries of EMR can take advantage of genomic data and apply the power of bioinformatics to local health problems without having to invest heavily in the technologies used to produce them, A dedicated Genomics and Health Research Fund for the region may help to break down financial barriers to encourage scientific research and development in the region.

The participants expressed enthusiasm for setting up National Biotechnology Commissions to address genomics policies at the national level and to contribute to the development of the above-mentioned national biotechnology strategy. A National Commission on Biotechnology (NCB) has been set up in Pakistan. The overall goal would be to help devise regulations to facilitate innovation in biotechnology. The NCB could help coordinate national biotechnology activities and policy, with activities including evaluation of biotechnology capacity (see above) to priority setting and public engagement. Accordingly, the NCB should have broad cross-sectoral representation in order to minimize the negative effects of inter-institutional rivalry. One of the main goals of the NCB (or equivalent national agency), following the above-mentioned national biotechnology survey, will be to help develop and adopt a national biotechnology strategy, perhaps as part of a long-term science and technology policy. Other important objectives are discussed below.

At the outset of the conference, participants voiced their concern about the low level of awareness (which goes beyond just *public *awareness) of biotechnology and genomics in the region, and that this lack of awareness may be the biggest barrier to advances in genomics. Public awareness and engagement can help change the pace of research, leading to increased opportunities for greater societal involvement for improved health care. Media can be used as a catalyst to raise community awareness for social beneficence, equity and justice in health care. Recently, in May 2004, science journalists from across the region met in Cairo to discuss the hurdles they face in science reporting. The hurdles identified at this meeting included bureaucracy and poor access to scientific research taking place in the region [26]. The meeting concluded with the creation of a provisional network of Arab science journalists that will aim to provide its members with training, skills and contacts, as well as promote the coverage of scientific issues from a development perspective. Similarly, public health specialists and scientists should also be encouraged to engage actively disseminate evidence based and correct information in disease prevention and control through media.

While it was agreed that science should be permitted to march forward, the participants emphasized that ethics, regulations and laws must keep up with the science [27]. For example, an important focus area of the NCB is that of intellectual property rights. The participants agreed that developing countries must build capacity and knowledge to choose the best policy options to benefit from the international patent regime. Compulsory licensing under specific circumstances may be a good option for developing countries and national laws should permit compulsory licensing [28]. The forces of globalization must balance forces of protectionism, especially by the developed world, and both national and regional regimes should respect international patent law to take advantage of globalization. If developing countries are involved in international research collaborations, they should ensure that they obtain patents. In view of this, it is essential for developing countries to build capacity and training in patent law and learn how to formulate effective patents, and the NCB could play a key role in this effort.

Regional cooperation in science was given a boost this year, with the establishment of a network of science academies of the Organisation of Islamic Conference [29] at a meeting organized by the Third World Academy of Sciences. This formal network aims to provide the partner states with mutual support and supports discussion of the scientific aspects of common problems. It could help to build a unified approach to capacity building in science and technology within member states.

Health advances in developing countries have lagged behind those in the developed world. The rapid advance in genomics research in developed countries compared with the relatively slow progress of genomics R&D in developing countries threatens to create a North-South genomics divide in the coming years, which may enhance existing health inequities. With the appropriate emphasis on its health needs, incentives for public-private R&D partnerships, and a sound set of regulatory policies, the Eastern Mediterranean Region may well reap the benefits of genomics and biotechnology. The overall goal of the Genome Policy Executive Course, a WHO-University of Toronto initiative, was to help provide the impetus for cross-sectoral dialogue on genomics and health policy in the region. The internet-based opinion leaders' network is expected to foster dialogue to help achieve the objectives outlined by the participants of the course.

The participants and the organizers of the course felt strongly that the recommendations formulated at the course must be shepherded by individuals. People felt that progress could be achieved if individuals make a significant and concerted effort to ensure that these recommendations are fulfilled.

One way to spur action and maintain the momentum generated by this course is by coordinating the participants into a network within which they can continue to interact and share information and experiences. This internet-based network will be moderated in order to streamline discussions. A number of short-term projects are envisioned that could be coordinated by various expert members of the network. The results of the survey (table 4) administered to the participants during the course suggested that 80% of them had reliable access to internet and would be willing to spend 1–2 hours a week taking part in the discussion. The main objectives of the network, as identified by the participants in the survey, would be dissemination of information, exchange of ideas, maintaining inter-connectivity, consensus building through wide participation, and influencing policy and media. The network is now established and is being used by the participants to exchange information.

**Table 4:** Opinion leaders' network survey results in brief

Goals of network	• Dissemination of information
	• Exchange of ideas
	• Maintaining inter-connectivity
	• Consensus building through wide participation
	• Influencing policy and media
Access	• 41 (79%) no access issues – reliable connectivity from work
	• 3 needed some assistance with email access (internet connection at work; compensation for access; help to post responses)

Obstacles to participation	• 47 (92%) identified lack of time due to professional responsibilities
	• 98% of those with connectivity willing to dedicate 1 hour a week to the network
	• 3 people identified lack of connectivity as a barrier

## Conclusions

The meeting concluded with a set of recommendations for the EMRO and Member States (table 5) [30]. The recommendations were developed through consensus among the participants. The process of consensus development involved the following steps: (i) the recommendations were drafted based on the group presentations, (ii) any recommendations which the participants did not support were deleted (iii) recommendations that were missing but deemed to be important were added (iv) the final list was scrutinized to sharpen language and consolidate points where possible.

**Table 5:** Recommendations

Recommendations for the Eastern Mediterranean Regional Office
The workshop recommends that the Regional Director EMRO may be requested to address the governments at the highest level for actively considering the proposals of this workshop and for giving priority attention to genomics for health and health biotechnology. The political leadership may be provided effective advocacy material, with special reference to its link with poverty alleviation, public health objectives, and need for transfer (and internalization) of technology.
EMRO and Organization of the Islamic Conference Standing Committee for Science and Technology (COMSTECH), and possibly other groups should provide coordination and networking among national biotechnology bodies (see below) and coordinators to exchange information, expertise, training, and Regional cooperation in production and utilization of health biotechnology.
EMRO, in collaboration with member states and their national biotechnology bodies, should coordinate a national survey/inventory/situation analysis/needs assessment of health biotechnology innovation systems, including scientific and management capacity, government policies, legislation and regulations, intellectual property policies, private sector activity, and strengths/weaknesses, opportunities and threats.
EMRO, in collaboration with COMSTECH and member states, should develop a proposal/feasibility study for a Regional Genomics and Health Research Fund emphasizing both peer-reviewed research and capacity strengthening.
Recommendations for Member States
Each member state should create an effective National Commission on Genomics, Biotechnology and Health, if this function has not otherwise been established, including a coordinator who will serve as the focal point for this activity. The membership should be multisectoral and include youth, women, and civil society. The focus should include ethical issues.
Based on evidence from the national survey described above, governments of member states should develop and adopt, at the highest level, a national biotechnology strategy.
The National Commission on Biotechnology should develop programs of public awareness and engagement. Important "publics" here include media and religious leaders as well as the public at large. The discussion should include ethical issues.
The National Commission on Biotechnology should encourage academic institutions including schools and universities, to include health biotechnology topics within their curricula and create specialized programs and degrees where appropriate. There should be particular emphasis on ICT and bioinformatics.
The National Commission on Biotechnology, in collaboration with the relevant ministries, should develop a plan to integrate genetic and genomics products (including diagnostics, vaccines, therapies, and other genomic priorities), within the health system and public health programs. The emphasis should be on accessibility and equity to improve the health of the poor.
Recommendations for Individuals
There is a need for strong personal commitment to strengthen the initiative on genomics and biotechnology to improve health and well-being of people in the EMRO Region. Workshop participants, as well as other concerned individuals, should are therefore encouraged to actively engage in the implementation of these recommendations.

The participants felt the need for EMRO to:

a) Request regional governments and policy makers at highest level to give priority to genomics for health and health biotechnology

b) Develop linkages with Organization of Islamic Conference Standing Committee for Science and Technology (COMSTECH) and other international partners to build Regional networking and cooperation for developing and utilizing health biotechnology.

c) In collaboration with Member States undertake a national survey/situation analysis/needs assessment of health biotechnology innovation systems including resource capacities, government policies (legislation, regulations intellectual property policies and private sector activity.

d) In collaboration with COMSTECH initiate a research grant for applied (health) genomics and biotechnology

The participants agreed that each member state would benefit from creating effective National Commissions on Biotechnology (NCB) to develop national biotechnology strategies. NCB should; a) develop national priorities, programmes and guidelines aimed at raising public education and awareness and b) collaborate with civil sectors to develop plans to integrate genetic and genomic products (including diagnostics, vaccines, therapies and other genomic priorities within the health systems and public health programmes and c) build capacities for utilization and access of health biotechnology to the needy and e) ensure ethical safeguards against unwanted harm and exploitation and improve equity to improve health of the poor. The participants also felt that there was a need for strong personal commitment at individual level by experts and key actors to engage actively in the implementation of the recommendations to strengthen the initiative on genomics and biotechnology to improve the health and well-being of people in EMR. One concrete outcome of the workshop that may contribute to strengthening capacity in the region is the joint agreement between WHO/EMRO and the University of Toronto Joint Centre for Bioethics to provide scholarships to the Master of Health Sciences Program at the JCB. Furthermore, there seems to be growing need to establish a Regional Health Biotechnology Network for EMR, and WHO-EMRO is now planning to hold a Regional meeting to discuss this in Iran in July 2004.

WHO-EMRO has initiated follow-up of these recommendations. Ministers of Health from the region, at the 50^th ^Regional Committee Meeting held in October 2003, urged Member States to establish national bodies for genomics and biotechnology to formulate strategic vision for creating public awareness and for developing biotechnology for equitable health care in the region.

Recently, a paper on harnessing genomics and biotechnology for public health was presented at the EM 28th Regional Consultative Committee Meeting (RCC) held in Cairo in April of this year. The recommendations in the paper were derived from those developed at the workshop. The paper will be presented at this year's Regional Committee Meeting to be held in October 2004.

The methods and recommendations outlined in this paper demonstrate progress in bringing genomics and biotechnology to the forefront of science policy in developing countries. Our procedure has now led to the formation of three regional networks – the two previous ones being the African Genome Policy Forum, which encompasses participants from 10 African nations, the Indian Genome Policy Forum. We have also now held a course for Latin America and the Caribbean in association with PAHO and the UN University in Venezuela May 23–26 2004, and a similar network is being created of participants of that course. The next one will be held in the Southeast Asian region, most likely based in Hong Kong. These regional genome policy networks will provide models to establish a Global Genome Policy Forum.

## Competing interests

The author(s) declare that they have no competing interests.

## Authors' contributions

TA drafted the manuscript; PAS and ASD conceived of the course, and all authors participated in its design and coordination. All authors revised the manuscript for critical content and approved the final draft.
